# Continuous Real-Time Motility Analysis of *Acanthamoeba* Reveals Sustained Movement in Absence of Nutrients

**DOI:** 10.3390/pathogens10080995

**Published:** 2021-08-06

**Authors:** Allison Campolo, Valerie Harris, Rhonda Walters, Elise Miller, Brian Patterson, Monica Crary

**Affiliations:** Department of Microbiology, Alcon Research, LLC, Fort Worth, TX 76134, USA; allison.campolo@alcon.com (A.C.); valerie-1.harris@alcon.com (V.H.); rhonda.walters@alcon.com (R.W.); elise.miller@alcon.com (E.M.); brian.patterson@alcon.com (B.P.)

**Keywords:** keratitis, *Acanthamoeba*, movement, velocity

## Abstract

*Acanthamoeba* keratitis is a serious ocular infection which is challenging to treat and can lead to blindness. While this pathogen is ubiquitous and can contaminate contact lenses after contact with water, its habits remain elusive. Understanding this organism’s natural behavior will better inform us on how *Acanthamoeba* colonize contact lens care systems. *Acanthamoeba* trophozoites were allowed to adhere to either a glass coverslip or non-nutrient agar (NNA) within a flow cell with nutrients (*Escherichia coli* or an axenic culture medium (AC6)) or without nutrients (Ringer’s solution). Images were taken once every 24 s over 12 h and compiled, and videos were analyzed using ImageJ Trackmate software. *Acanthamoeba* maintained continuous movement for the entire 12 h period. ATCC 50370 had limited differences between conditions and surfaces throughout the experiment. Nutrient differences had a noticeable impact for ATCC 30461, where *E. coli* resulted in the highest total distance and speed during the early periods of the experiment but had the lowest total distance and speed by 12 h. The Ringer’s and AC6 conditions were the most similar between strains, while *Acanthamoeba* in the *E. coli* and NNA conditions demonstrated significant differences between strains (*p* < 0.05). These results indicate that quantifiable visual tracking of *Acanthamoeba* may be a novel and robust method for identifying the movement of *Acanthamoeba* in relation to contact lens care products. The present study indicates that *Acanthamoeba* can undertake sustained movement for at least 12 h with and without nutrients, on both rough and smooth surfaces, and that different strains have divergent behavior.

## 1. Introduction

*Acanthamoeba* keratitis (AK) is a serious ocular infection that can occur as a result of contact lens contamination with tap water, non-compliance with lens wear and care, and/or ineffective lens cleaning solutions [1]. Outbreaks of AK have occurred or are ongoing in the United States and the United Kingdom [2,3,4,5]. While demonstrating *Acanthamoeba* disinfection efficacy is not currently required for commercial contact lens care products, the International Standards Organization is currently examining the protocols necessary to add *Acanthamoeba* disinfection efficacy to the requirements for commercially produced contact lens care products [6,7]. However, due to the unique and specific nature of AK, *Acanthamoeba* research has been largely limited to pathogenicity and contact lens care disinfection, with work also performed on endosymbionts and intracellular viruses, which has led to limited understanding of this organism’s basic motility and how this motility extends to health care fields such as contact lens care solutions. Therefore, we here aim to outline a novel method for examining *Acanthamoeba* responses in real time to materials, and examine the ability of *Acanthamoeba* to survive and remain motile in both nutrient-rich and nutrient-poor conditions.

Live-cell time-lapse imaging is a burgeoning field of research that has risen alongside advancements in bright-field and fluorescent microscopy. These new tools have been used to successfully track and quantify the movements of many different microscopic populations, such as human neural stem cells, breast adenocarcinoma cells, and *Drosophila* [8,9,10]. These techniques have also recently been used to quantify the cytopathic effects on *Acanthamoeba* motility [11] after infection with a giant virus [12] and during exposure to an electric field [13]. However, to our knowledge, this technology has never been used to examine multiple *Acanthamoeba* strains for movements on various surfaces and in varying nutritional states for a period as long as 12 h.

As the contact lens care community continues to improve amoebicidal disinfection efficacy and take steps to understand how *Acanthamoeba* may overcome ineffective contact lens care solutions, or how patient non-compliance with prescribed contact lens care regimens might lead to *Acanthamoeba* colonization of contact lens systems, many aspects of *Acanthamoeba* biology remain elusive. Therefore, this study seeks out to demonstrate the strength of live time-lapse *Acanthamoeba* imaging as a quantitative method to understand *Acanthamoeba* behavior and movements during the control conditions commonly used in ocular *Acanthamoeba* research [14,15]. The control conditions examined here are combinations of commonly-used surfaces and solutions: glass with Ringer’s solution, glass with *E. coli*, glass with an axenic culture medium, and non-nutrient agar with Ringer’s solution. By determining the basic behaviors of this organism, contact lens system manufacturers will be able to communicate safe practices and best products to doctors and patients by improving our understanding of which product aspects provide the most infection prevention and/or disinfection efficacy.

## 2. Results

*Acanthamoeba* trophozoites were allowed to adhere to the glass coverslip or a coverslip covered in NNA for 30 min before the beginning of image recording. The initial *Acanthamoeba* suspension solutions were exchanged with the experimental solutions prior to initiating recording. *Acanthamoeba* were recorded for 12 h in either ¼ Ringer’s solution (Ringer’s), ¼ Ringer’s solution containing *E. coli* or an axenic culture medium (AC6). Movements over 30 min increments were averaged and compared.

### 2.1. Total Distance

**Figure 1:**
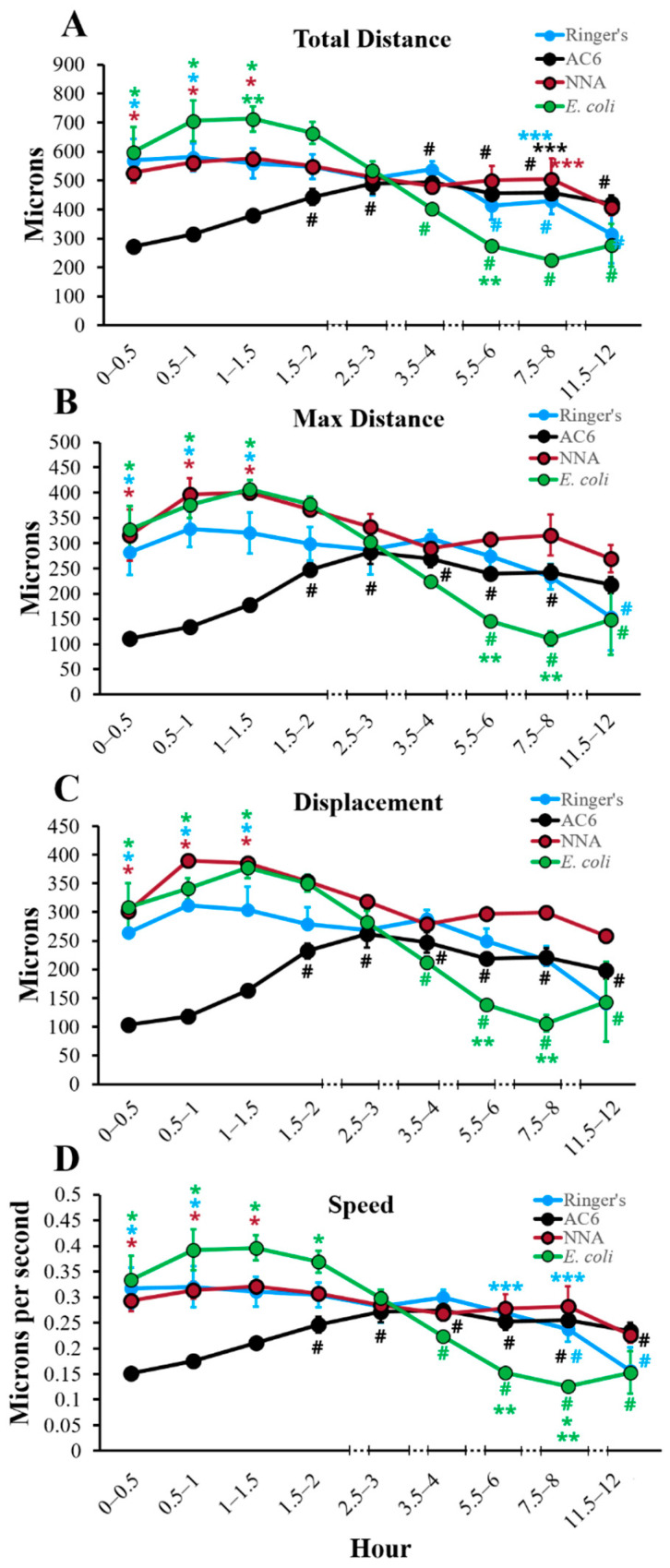
Quantification of movement of the ATCC 30461 (*A. polyphaga*) strain over 12 h in four different control conditions. (**A**) total distance, (**B**) max distance, (**C**) displacement, and (**D**) speed. * *p* < 0.05 vs. AC6, ** *p* < 0.05 vs. NNA, *** *p* < 0.05 vs. *E. coli*, and # *p* < 0.05 vs. 0–0.5 time point within the same condition. Color of each statistical symbol corresponds to the stated comparison. n = 3 replicates per group/time point/strain, and 20–200 tracks per replicate.

**Figure 2:**
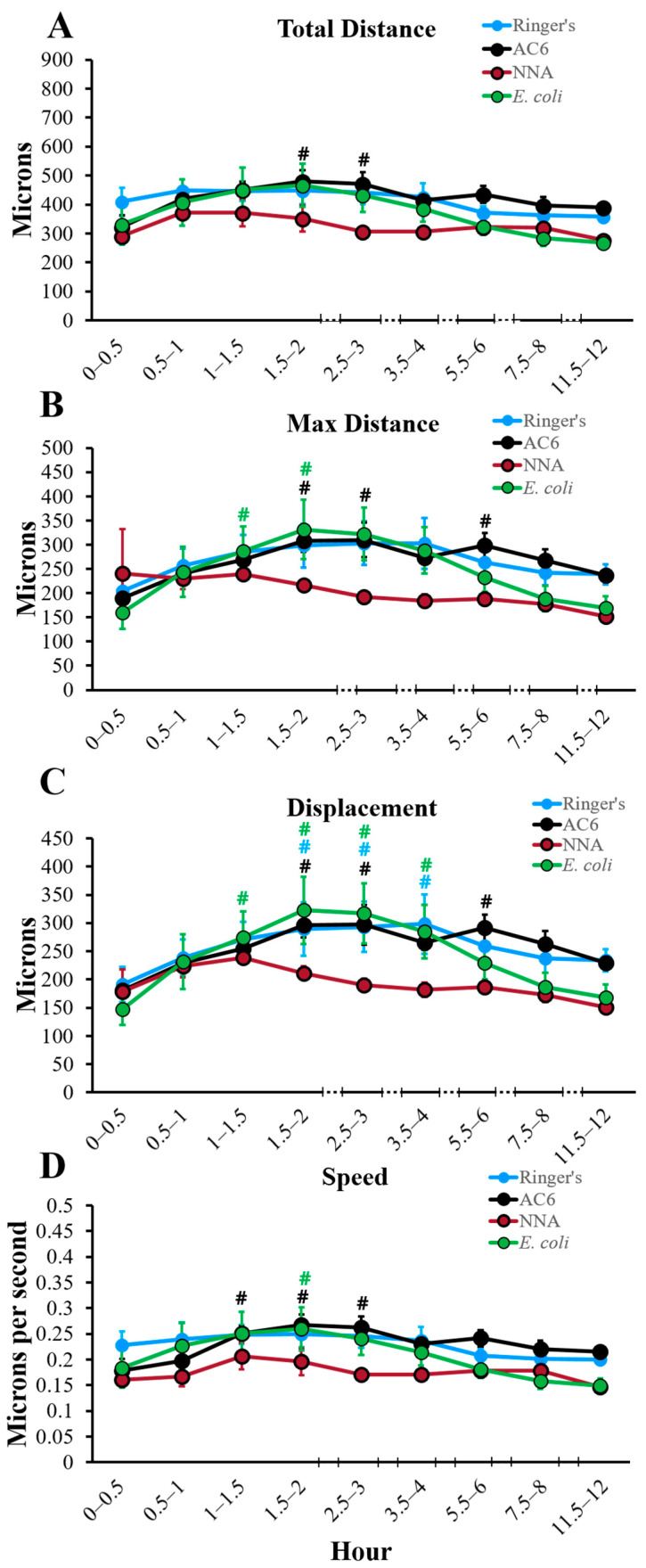
Quantification of movement of the ATCC 50370 (*A. castellani*) strain over 12 h in four different control conditions. (**A**) total distance, (**B**) max distance, (**C**) displacement, and (**D**) speed. # *p* < 0.05 vs. 0–0.5 time point within the same condition. Color of each statistical symbol corresponds to the stated comparison. n = 3 replicates per group/time point/strain, and 20–200 tracks per replicate.

**Figure 3:**
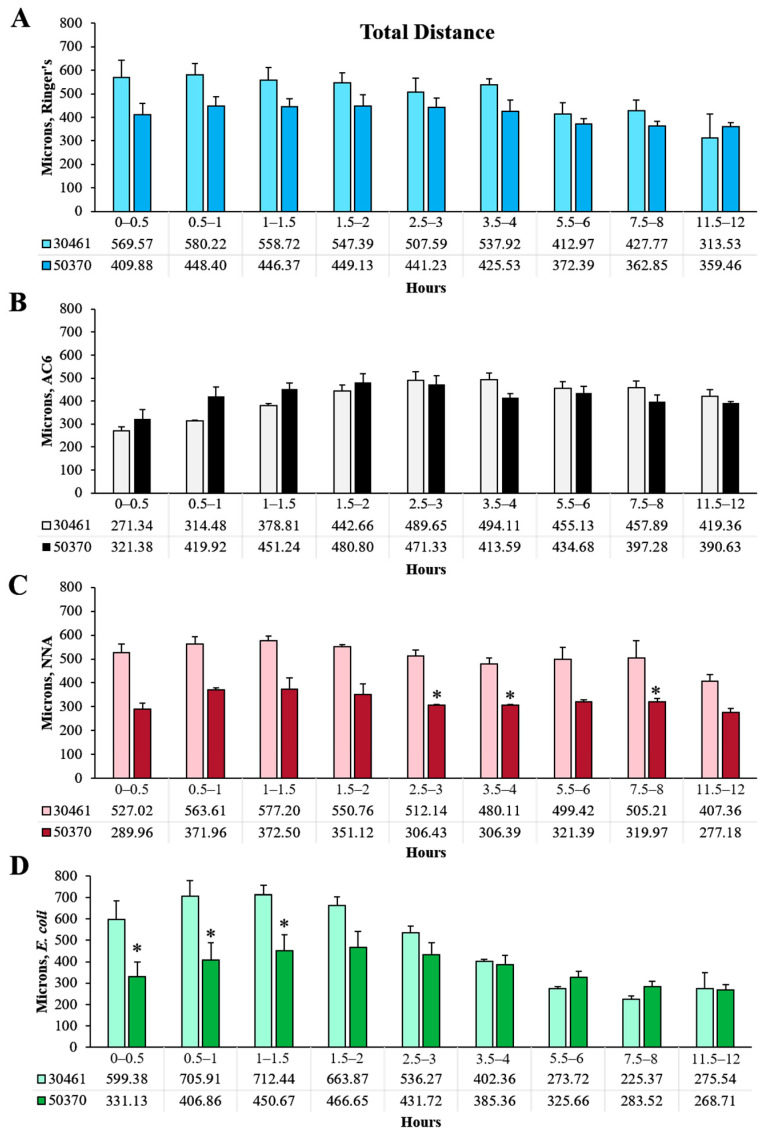
Comparison between strains within each condition and each time point of total distance of *Acanthamoeba* movement. (**A**) Ringer’s solution, on glass; (**B**) an axenic culture medium (AC6), on glass; (**C**) non-nutrient agar (NNA) with Ringer’s solution; (**D**) *E. coli* suspended in Ringer’s solution, on glass. * *p* < 0.05 vs. ATCC 30461. n = 3 replicates per group/time point/strain, and 20–200 tracks per replicate.

Total distance traveled by each amoeba, which describes the entire distance achieved within the 30 min time frame, within each replicate, was quantified and analyzed (Figure 1A, Figure 2A and Figure 3). Within total distance, it is evident that a consistent distance by all amoeba is traveled for the majority of the time in all of the conditions tested. This is most clearly demonstrated within ATCC 50370 for all four conditions tested, where no combination of surface/nutrients resulted in statistically significant differences in total distance traveled (Figure 2A). For ATCC 30461, total distance with NNA closely resembled the Ringer’s condition over the course of the 12 h. *E. coli* demonstrated the highest total distance between 0.5 and 2 h followed by a dramatic decline, resulting in a statistically lower total distance compared to all other conditions at 7.5–8 h. AC6 had a significantly lower total distance to 1 h compared to the other 3 conditions for ATCC 30461. Three conditions (AC6, Ringer’s and *E. coli*) demonstrated a significant difference in total distance at 11.5–12 h versus the start point for ATCC 30461, where AC6 had a higher total distance at the end of the experiment compared to the start, whereas the Ringer’s and *E. coli* conditions were significantly lower. For the ATCC 30461 strain, the greatest total distance at any time point was 712 µm in the *E. coli* condition at 1–1.5 h, compared to 580 µm in the Ringer’s condition at 0.5–1 h, 577 µm in the NNA condition at 1–1.5 h, and 494 µm in the AC6 condition at 3.5–4 h, indicating that peak total distance was achieved at early time points (before 1.5 h) for all conditions except AC6. In contrast, ATCC 50370 achieved peak total distance at 1.5–2 h in AC6 with 481 µm, which was 231 µm less total distance traveled than the highest total distance achieved by ATCC 30461. The total distances for the Ringer’s, NNA and *E. coli* conditions were also generally higher in the ATCC 30461 strain versus 50370 (Figure 3), with statistical differences being notable in the *E. coli* condition between 0 and 1.5 h. Regarding the whole 12 h period of the ATCC 50370 trophozoites in the *E. coli* and NNA conditions, these amoeba only traveled 76% and 63% as far as those in the same conditions, respectively, in the ATCC 30461 strain. Interestingly, the Ringer’s and AC6 condition distances were highly similar between the two strains, with no statistically significant differences between the two strains during the 12 h period. This is more interesting given the dramatic difference in response to the *E. coli* condition between the strains.

### 2.2. Max Distance

**Figure 4:**
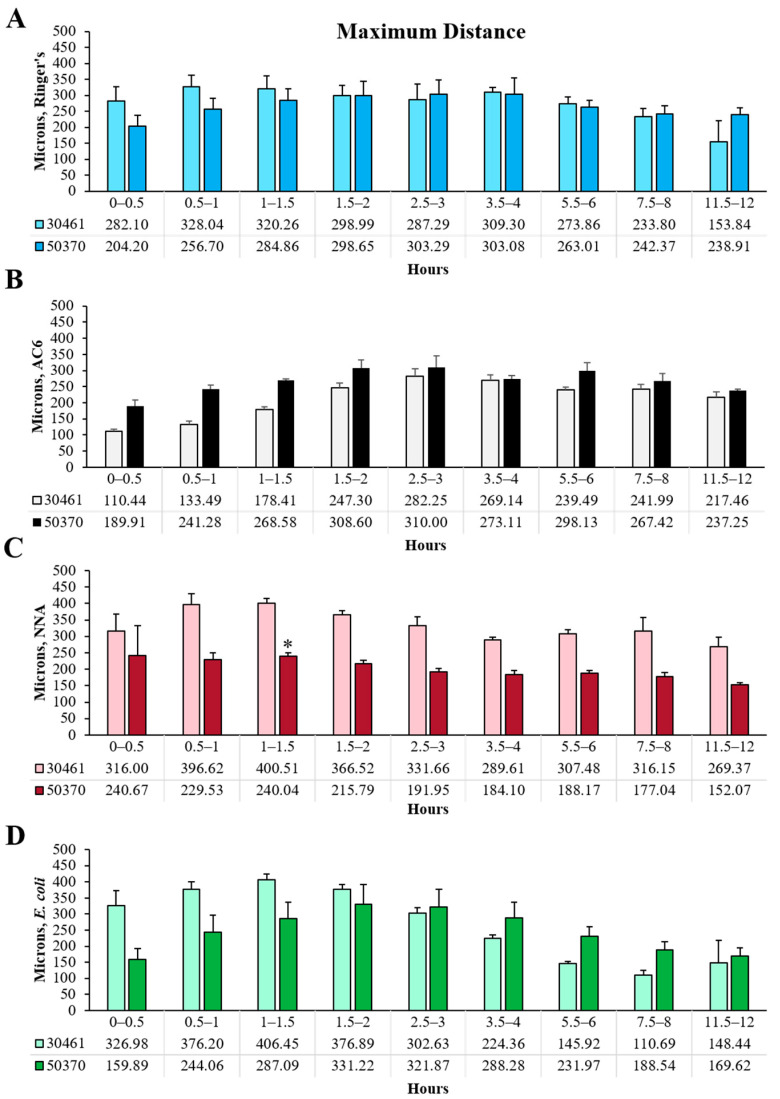
Comparison between strains within each condition and each time point of max distance of *Acanthamoeba* movement. (**A**) Ringer’s solution, on glass; (**B**) an axenic culture medium (AC6), on glass; (**C**) non-nutrient agar (NNA) with Ringer’s solution; (**D**) *E. coli* suspended in Ringer’s solution, on glass. * *p* < 0.05 vs. ATCC 30461. n = 3 replicates per group/time point/strain, and 20–200 tracks per replicate.

Max distance (Figure 1B, Figure 2B and Figure 4), which describes the greatest linear distance from the point of origin within any given timeframe, yielded highly similar results to total distance. In ATCC 30461, all conditions gradually increased their max distance to 1.5 h (3 h for AC6) before generally declining to 12 h. In contrast, ATCC 50370 maintained an extremely gradual incline over time until 2 h, followed by a plateau until 3 h, and a very gradual loss of max distance to 12 h, despite the extreme differences in nutrient availability and surface roughness between these four conditions. These gradual changes in max distance resulted in only one statistical difference between the strains, with 50370 having a significantly lower max distance at 1–1.5 h in NNA compared to the same time point and condition in the ATCC 30461 strain. The max distance highlights the impact of observing a population of organisms where a large number of trophozoites exhibit a variety of responses that do not lend themselves to statistical differences and only trends can be observed. Like total distance, ATCC 50370 maintained a largely steady max distance traveled throughout the experiment while ATCC 30461 was more sensitive to its nutrient and surface roughness environment. When summarizing the entire 12 h, it is evident that the two strains demonstrated remarkably similar max distance results, with the only exception being the 50370 NNA condition possessing approximately 61% of the entire max distance of the 30461 NNA condition. Thus, these results indicate that these two strains forage for nutrients in a similar pattern compared to their starting position.

### 2.3. Displacement

**Figure 5:**
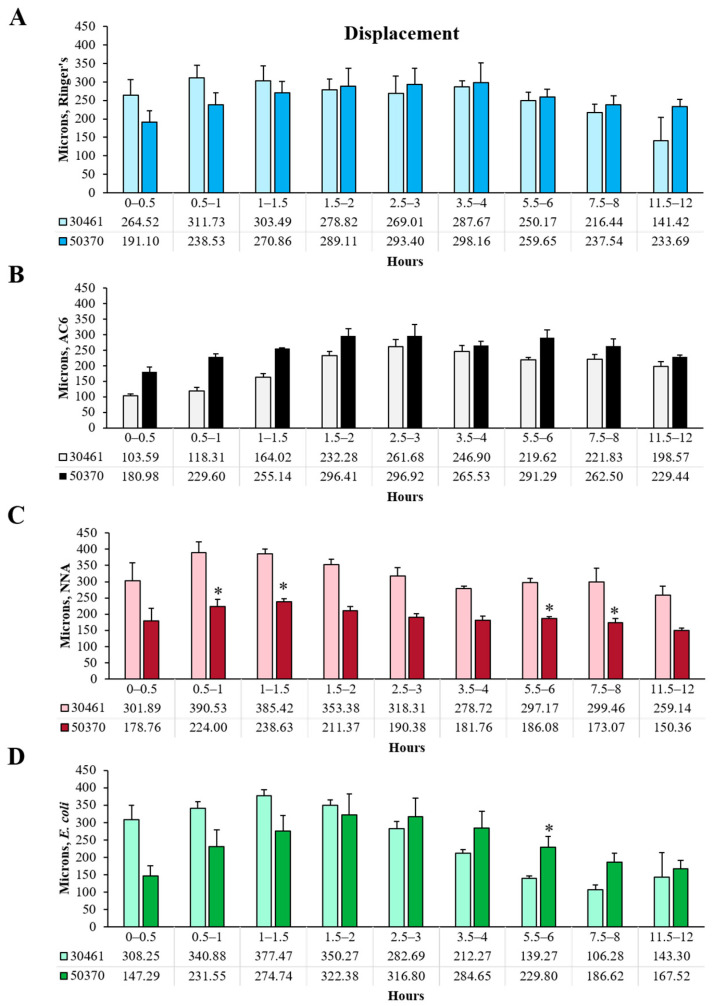
Comparison between strains within each condition and each time point of displacement of *Acanthamoeba* movement. (**A**) Ringer’s solution, on glass; (**B**) an axenic culture medium (AC6), on glass; (**C**) non-nutrient agar (NNA) with Ringer’s solution; (**D**) *E. coli* suspended in Ringer’s solution, on glass. * *p* < 0.05 vs. ATCC 30461. n = 3 replicates per group/time point/strain, and 20–200 tracks per replicate.

Displacement was quantified to determine the linear distance between the start and end points for each amoeba within each half-hour analyzed (Figure 1C, Figure 2C and Figure 5). Overall, displacement, total distance and max distance demonstrated similar trends, indicating that amoeba traveled in a generally forward direction during any one time point, instead of doubling back on their path. Similar to the results demonstrated in Figure 1, Figure 2, Figure 3 and Figure 4, subpanel 1C demonstrates that within the ATCC 30461 strain, the Ringer’s, *E. coli* and NNA conditions all peaked between 0.5 and 1.5 h, and decreased steadily from there. All of these conditions started significantly higher than AC6 at the baseline (*p* < 0.05), but only *E. coli* and AC6 were both significantly different at 11.5–12 h versus their own 0–0.5 h baseline (*p* < 0.05). Specifically, AC6 displacement was significantly higher from its baseline starting at 1.5 to 12 h (*p* < 0.05). Within the ATCC 50370 strain, all four conditions had similar displacement to 1.5 h, although NNA trended with lower displacement through the end of the experiment. No statistical differences were observed between the conditions. When compared between strains (Figure 5), only the NNA condition generated statistically lower displacement in ATCC 50370 than ATCC 30461 between 0.5–1.5 h and 5.5–8 h (*p* < 0.05). Thus, similar to the max distance results (in both µms and in overall trend), the displacement between the two strains was remarkably similar, indicating both strains’ ability to continue forward movement regardless of nutrient availability.

### 2.4. Speed

**Figure 6:**
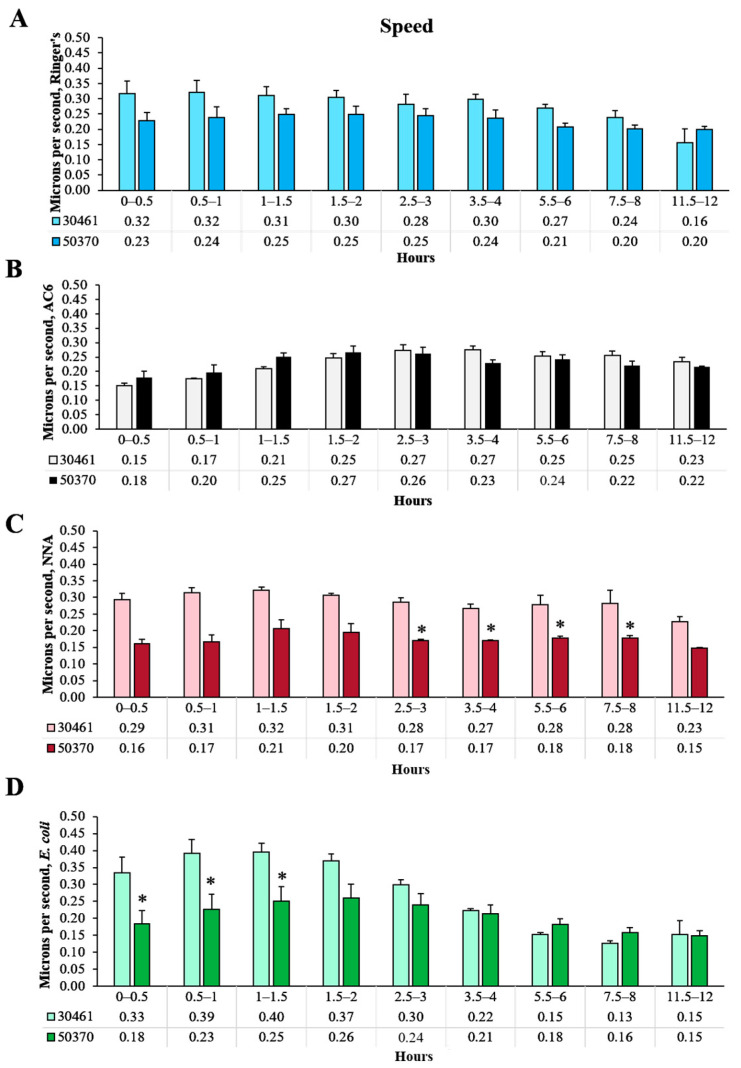
Comparison between strains within each condition and each time point of speed of *Acanthamoeba* movement. (**A**) Ringer’s solution, on glass; (**B**) an axenic culture medium (AC6), on glass; (**C**) non-nutrient agar (NNA) with Ringer’s solution; (**D**) *E. coli* suspended in Ringer’s solution, on glass. * *p* < 0.05 vs. ATCC 30461. n = 3 replicates per group/time point/strain, and 20–200 tracks per replicate.

Acanthamoeba speed was also measured in microns traveled per second (Figure 1D, Figure 2D and Figure 6). As a function of total distance, speed closely resembles the total distance results demonstrated in Figure 1, Figure 2 and Figure 3. Overall within ATCC 30461, *E. coli* demonstrated a higher speed trend with a peak of 0.40 µm/s at 1.5 h compared to the other three conditions, and then had a steady decline from there to 12 h (Figure 1D). AC6 within this same strain started significantly slower (*p* < 0.05) compared to the other three conditions at approximately 0.15 µm/s and then had comparable speed to the Ringer’s and NNAS conditions by 3 h through to the end of the experiment. Indeed, the AC6 condition within ATCC 30461 was the only strain/condition combination to have a higher rate of speed at 12 h versus the baseline, whereas the Ringer’s and *E. coli* conditions had a significantly lower rate of speed at the end of the experiment versus start point. Within the ATCC 50370 strain, no significant differences in speed were seen between any conditions at any time points. Overall, the ATCC 30461 conditions demonstrated the greatest loss of speed by 12 h with only 50% and 46% of baseline speed for the Ringer’s and *E. coli* conditions, respectively. ATCC 50370 speed remained fairly consistent at 12 h with 88%, 121%, 91%, and 81% of speed compared to at 0–0.5 h in the Ringer’s, AC6, NNA, and *E. coli* conditions, respectively. Thus, the ATCC 50370 strain is able to maintain almost its full starting speed by 12 h, with or without nutrients, on both smooth and rough surfaces.

## 3. Discussion

*Acanthamoeba* keratitis (AK) is a disease which is notoriously difficult to diagnose and extremely challenging to treat or manage, and may result in devastating effects on eye function [1,16]. Unfortunately, the outbreaks in the United States and United Kingdom [2,3,4,5] indicate that this infection persists in developed countries, and highlight the challenges of properly maintaining disinfected reusable contact lenses [17]. Indeed, contact lens care cases, even those used by asymptomatic lens wearers, have been found to have contamination by viable bacterial colonies and *Acanthamoeba* trophozoites or cysts [18]. While there is currently no standard requirement for demonstration of *Acanthamoeba* disinfection by contact lens care products, the International Standards Organization is currently assessing *Acanthamoeba* disinfection procedures and outcomes in order to add this organism to the required testing for all products [6,7]. However, very little is currently known about real-time *Acanthamoeba* behavior and how that behavior may translate to contact lens systems. Thus, in this study, we expanded a recently-described method [11,12,13] with the goal of providing specific answers as to how *Acanthamoeba* behave for future extrapolation into *Acanthamoeba* activity in the context of a potential contamination event in a contact lens care system, which could lead to an ocular infection. The present study takes the first steps in this endeavor: understanding how these amoeba behave in a potential overnight period, using several different types of commonly used “control” settings in ocular *Acanthamoeba* research, with and without nutrients provided.

The four different control conditions used here produced important information regarding the speed, distance, and displacement of *Acanthamoeba* when the amoeba is given a smooth environment with and without nutrients (AC6, *E. coli,* and Ringer’s conditions), and an agar surface without nutrients (NNA with Ringer’s solution). Further, two of the most common strains of *Acanthamoeba*, *A. castellanii* (ATCC 50370) and *A. polyphaga* (ATCC 30461)*,* were used. Most notably, in the ATCC 50370 strain, distance, displacement, and speed all remain remarkably consistent across the experiment regardless of surface, nutrient availability, or nutrient type. However, *Acanthamoeba* within the ATCC 30461 strain were slow to acclimate to the AC6 condition compared to ATCC 50370, taking until 3 h to achieve similarity in distance and speed compared to the other conditions. It is interesting to note that while *Acanthamoeba* were cultured in AC6, they were then harvested in Ringer’s solution. The slow movement of ATCC 30461 in AC6 indicates a poor return to normal movement between different solutions, despite being regularly exposed to both the Ringer’s and AC6 conditions as part of normal culturing. In contrast, ATCC 30461 showed the highest movement (total distance, max distance, displacement and speed) in *E. coli* despite coming from an axenic culture in the absence of a bacterial food source. The response of ATCC 30461 to a bacterial food source was a rapid forging event across a large area for several hours before slowing and decreasing distance as time went by. It is interesting that *Acanthamoeba* did not adapt similarly to AC6, suggesting a complex response based on the type of nutrients available. It is then surprising that *Acanthamoeba* did not greatly reduce movement in AC6 as foraging was required to obtain nutrients. Overall, this study demonstrates that amoeba will move in a similar forward direction despite having readily available nutrients at their current location, even if the movement is slightly slower and is nutrient type dependent. These differences between strains allude to strain-dependent proclivities of what makes migration more or less important to an amoeba, which is similar to previous studies indicating that *Acanthamoeba* move robustly in all directions regardless of density or presence of folate [19]. While the natural assumption may be that both strains would be chiefly interested in conservation of energy, ATCC 50370 maintained its movement regardless of surface or nutrient availability. In contrast, ATCC 30461 utilized initial food availability to increase distance and speed when the bacterial nutrients were readily available but then decreased movement dramatically overtime. While bacteria were still visibly present at the end of 12 h, it is possible that the natural decrease in bacterial concentration as a result of consumption triggered ATCC 30461 to reduce movement in order to conserve energy in preparation for starvation conditions. This hypothesis is further supported by the final results for total distance, max distance, displacement, and speed all being nearly identical between the Ringer’s and *E. coli* conditions for ATCC 30461.

However, it is important to note that the Ringer’s condition produced highly similar results between the two strains. This is a condition that does not provide rough agar to walk on or nutrients to consume, but only a neutral fluid environment. The results of the Ringer’s condition indicate here that both strains, without any additional support, can continue moving for at least 12 h. This means *Acanthamoeba* could migrate on or around stored contact lenses and contact lens cases if left in tap water or in an ineffective disinfection solution in a normal overnight period. Even without nutrients, amoeba will migrate in forward progressive movements, not doubling back on their tracks and instead seeking out the most hospitable environment or surface for at least an entire 12 h period.

Quantitatively, we can use this information to understand how *Acanthamoeba* movement specifically relates to the potential for movement on or around contact lens cases or in used contact lens care products. Previous studies indicate that *Acanthamoeba* trophozoites in a control environment travel at a speed of approximately 12.1 µm/min (0.20 µm/s) [13], which is highly similar to the results we demonstrate here. In 12 h, at this rate of speed, an *Acanthamoeba* trophozoite can traverse approximately 40% of a contact lens, which is 22 to 25 mm across. In other words, if an amoeba contaminates a lens case (from tap water, ocean water, dirt, etc.), that amoeba can walk around the interior lens case within the overnight period if the contact lens care solution fails to inhibit motility. Further, the max distance and displacement values being so similar between each strain and condition indicate that in both nutrient-rich and nutrient-free environments, *Acanthamoeba* are likely to move in a generally forward direction from their starting point, and not stay in one place in a circular motion. Additionally, our results indicate that without an outright harmful environment, *Acanthamoeba* are unlikely to encyst, even in a nutrient-free medium, within 12 h. Indeed, at the 12 h time point, no condition or strain demonstrated a lack of movement, indicating that amoeba within all of these condition and strain combinations would continue moving past the end of the examination period. That is, lack of nutrients alone is not sufficient to trigger encystment within 12 h.

In conclusion, this methodology is applied for the first time to quantitatively define and measure multiple control-type conditions and strains of *Acanthamoeba*, with the goal of better understanding how this organism might interact with used contact lenses, and how contact lens care solutions may best protect against *Acanthamoeba*. By being able to create an hour-by-hour study of *Acanthamoeba* behavior, we can begin to discern *Acanthamoeba* movement on contact lenses, with and without contact lens care solutions, and we may better understand how commercially available disinfection protocols will best eliminate the threat of *Acanthamoeba* keratitis. The results of the present study demonstrate for the first time that within any of the multiple control-type conditions used to mimic the natural *Acanthamoeba* condition, amoeba can sustain continuous movement for at least 12 h with and without nutrients, and in both smooth and rough surfaces.

## 4. Materials and Methods

### 4.1. Acanthamoeba Trophozoite Culturing

As previously described [14], an axenic culture medium (AC6; containing 20 g biosate peptone, 5 g glucose, 0.3 g KH_2_PO_4_, 10 µg vitamin B12, and 1glass5 mg l-methionine per liter of distilled deionized water) was used to axenically produce *Acanthamoeba* trophozoites. AC6 was adjusted to a pH of 6.6–6.95 with 1 M NaOH and autoclaved at 121 °C for 20 min before being stored at room temperature for use within 3 months. One-quarter Ringer’s solution was used to harvest organisms. *Acanthamoeba* strains were obtained from American Type Culture Collection (ATCC, Manassas, VA, USA). Strains used were ATCC 30461 (*Acanthamoeba polyphaga*, Group T4, isolated from human eye infection, Namibia or South Africa, 1973) and ATCC 50370 (*Acanthamoeba castellanii*, Group T4, isolated from human eye infection, New York, NY, 1978). These strains belong to the T4 genotype, which is the most commonly associated genotype with *Acanthamoeba* keratitis [20,21,22]. *Acanthamoeba* trophozoites were subcultured in axenic medium with the final 24 h of growth in fresh medium to promote uniform *Acanthamoeba* trophozoite proliferation prior to testing to ensure a homogenous population of trophozoites.

### 4.2. Acanthamoeba Flow Cell Suspension

**Figure 7:**
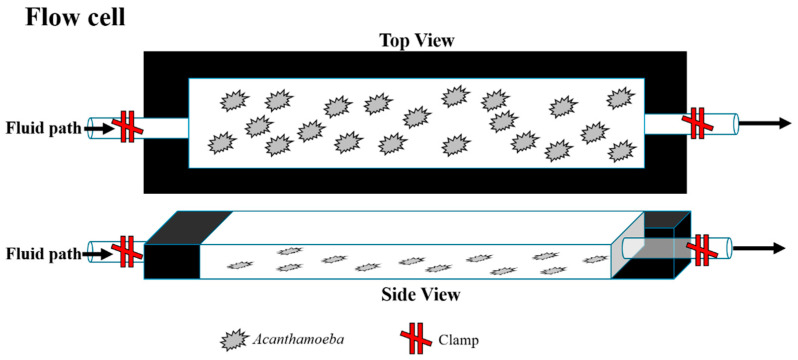
Schematic representation of flow cell apparatus. Top and side views of flow cells indicating layout of amoeba attachment to the bottom pane of the flow cell. *Acanthamoeba* suspended in control condition medium are inserted into the flow cell via the inward port, and both the entry and exit ports are clamped for the 12 h experimental period to create an environment without fluidic movement.

Sterile aluminum transmission flow cells (Biosurface Technologies Corporation, Bozeman, MT, USA) were assembled with glass coverslips. For NNA surface conditions, a thin layer of NNA was added to the glass coverslip during flow cell assembly and allowed to solidify. *Acanthamoeba* suspensions (7.5 × 10^3^ cells/mL) in ¼ Ringer’s solution were added through the ports of the flow cell (Figure 7). The flow cell ports were clamped shut to stop the flow of *Acanthamoeba* suspension and *Acanthamoeba* trophozoites were allowed to adhere to the glass coverslip or non-nutrient agar (NNA) surface of the flow cell for 30 min prior to experiment start. After 30 min, the nutrient-free (Ringer’s condition) or nutrient-rich (AC6 or *E. coli*) solution was added to the flow cell. The solutions used were ¼ Ringer’s solution, ¼ Ringer’s solution containing 10^6^ CFU/mL *E. coli*, or AC6, as only control-type conditions common to *Acanthamoeba* research were used to examine the natural amoebic movements. To exchange the *Acanthamoeba* suspension fluid for the experimental solutions, 4 mL of each experimental solution was slowly added through the ports of the flow cell, taking care not to disturb adhered amoeba. The flow cell was then clamped closed at each end to create a closed system and no further fluid exchange occurred for the duration of the experiment. The apparatus was then attached to the microscope stage. Images were taken at 4× magnification using a Nikon Eclipse Ti-U Microscope (Nikon, Tokyo, Japan) every 24 s for hours 0 through 2, 2.5–3, 3.5–4, 5.5–6, 7.5–8, and 11.5–12 h, and the images were subsequently put together into a video format using NIS Elements AR 3.2. Each field contained ≥100 amoeba.

### 4.3. Acanthamoeba Track Analysis

**Figure 8:**
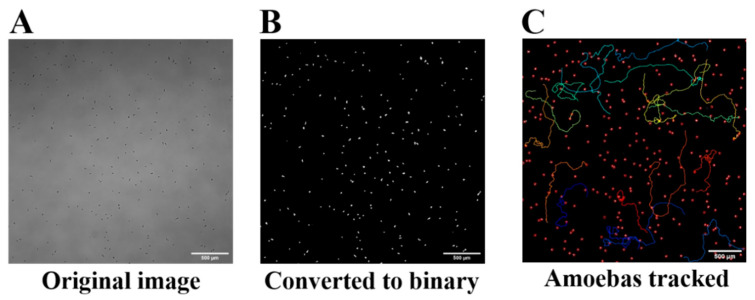
Representative images of track analysis process. (**A**) Bright-field microscope image taken in original greyscale. (**B**) Same image converted to binary, where *Acanthamoeba* appear as white organisms on a black background. (**C**) Same image with select tracks shown (not all tracks present), where ImageJ assigns a unique color for each individual track. Magnification = 4×; scale bar = 500 μm.

Videos were recorded, using bright-field microscopy, in grayscale. Using ImageJ, videos (Figures S1 and S2) were converted into a high-contrast, binary format for analysis (Figure 8). Briefly, background was subtracted utilizing rolling ball 50 parameters and contrast enhanced by 0.1%. Automatic thresholding as determined by ImageJ was used to convert greyscale images into binary. Non-amoebic artifacts were removed utilizing fill and clear functions within ImageJ as needed. Videos were analyzed using ImageJ Trackmate software to quantify amoeba movement, using estimated blob sizes of 35 μm, and allowing for 40 μm movements between frames. Trackmate was set to allow 4 frame gap closures if movement was less than 100 μm for the identified cell, and tracks representing less than 95% of the frames captured were discarded. Three independent replicates were recorded for each condition per strain, with between 20 and 200 trackable amoeba in each replicate. Tracks were considered unusable if amoeba walked on or off the field of view at any time during recording. total distance, max distance, speed, and displacement were calculated for each organism track in each replicate.

### 4.4. Tracking Algorithms

**Figure 9:**
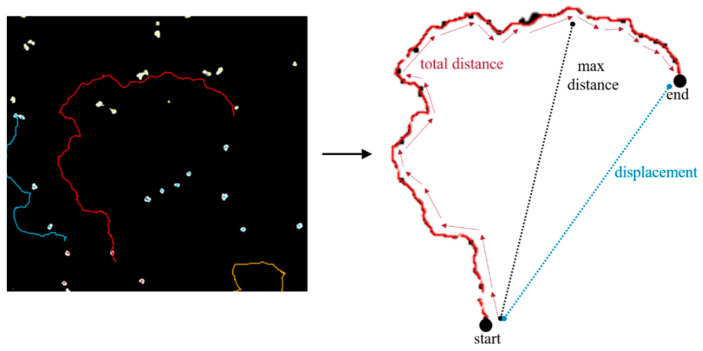
Representative image of track algorithms. Left, original tracked image. Right, representative parameters describing how total distance, max distance, and displacement are determined.

Please reference the following parameters in Figure 9, as an example of each calculation [23]. Total distance traveled is how much a trophozoite walked over the half-hour period, in total. It is calculated as $\;total\;distance\;traveled=\overset{}{\underset{i}{\sum }}d_{i,i}+1$, where *d_i,i_* + 1 is the distance from one spot to the next spot in the track.

Max distance traveled represents the distance to the furthest point of the track, with respect to the first spot of the track (at the beginning of the half-hour). This is computed by finding the straight-line distance between these two points. This is calculated as $max\;distance\;traveled=Max_{i.j}\left(d_{ij}\right)$, where *d_ij_* is the distance from any spot *i* to any spot *j* in the track.

Mean speed (not to be confused with mean straight-line speed) is defined as the total distance divided by the total track time. This is calculated as $mean\;speed=\frac{total\;distance}{total\;track\;time}$, to determine the number of microns traveled per second.

Finally, displacement is the measured distance between the last spot of the track and the first spot of the track in time.

### 4.5. Statistics

The data from each amoeba track within a replicate were averaged, and then each replicate averaged for a sample size of 3 for each time point, condition, and strain. The mean and standard error were calculated from these replicates. Statistical analysis was completed via one-way ANOVA for comparison between strains within each condition and time point (Figure 2, Figure 4, Figure 6 and Figure 8). Two-way repeat-measures ANOVA was used for comparisons within time and between conditions (Figure 1, Figure 3, Figure 5 and Figure 7). Both analyses were followed by Tukey’s post hoc multiple comparisons test, and significance was set at *p* < 0.05.

## Data Availability

The data presented in this study are available upon request from the corresponding author. The data are not publicly available due to commercial interests.

## Supplementary Materials

The following are available online at https://www.mdpi.com/article/10.3390/pathogens10080995/s1, Figure S1. Example time-lapse video of *Acanthamoeba* movement from 0 to 2 h, with images taken every 24 s. Figure S2. Example time-lapse video of *Acanthamoeba* movement from 0 to 2 h, with images taken every 24 s, and select tracks shown. For clarity, not all tracks included in video.

## References

[1] Szentmary N., Daas L., Shi L., Laurik K.L., Lepper S., Milioti G., Seitz B. (2019). Acanthamoeba keratitis—Clinical signs, differential diagnosis and treatment. J. Curr. Ophthalmol..

[2] Carnt N., Hoffman J.J., Verma S., Hau S., Radford C.F., Minassian D.C., Dart J.K.G. (2018). *Acanthamoeba* keratitis: Confirmation of the uk outbreak and a prospective case-control study iden-tifying contributing risk factors. Br. J. Ophthalmol..

[3] Verani J.R., Lorick S.A., Yoder J.S., Beach M.J., Braden C.R., Roberts J.M., Conover C.S., Chen S., McConnell K.A., Chang D.C. (2009). National outbreak of acanthamoeba keratitis associated with use of a contact lens solution, united states. Emerg. Infect. Dis..

[4] Datta A., Willcox M.D., Stapleton F. (2021). In vivo efficacy of silver-impregnated barrel contact lens storage cases. Contact Lens Anterior Eye.

[5] Tu E.Y., Joslin C.E. (2010). Recent outbreaks of atypical contact lens-related keratitis: What have we learned?. Am. J. Ophthalmol..

[6] ISO 14729:2001/A1 (2010). Ophthalmic Optics–Contact Lens Care Products–Microbiological Requirements and Test Methods for Products and Regimens for Hygienic Management of Contact Lenses.

[7] ASC Z80, Parent Committee Meeting, February 27. https://www.thevisioncouncil.org/sites/default/files/ASCZ80_ParentCommitteeMinutes_February_27_2018_FINALMar19-2018.pdf.

[8] Piltti K.M., Cummings B.J., Carta K., Manughian-Peter A., Worne C.L., Singh K., Ong D., Maksymyuk Y., Khine M., Anderson A.J. (2018). Live-cell time-lapse imaging and single-cell tracking of in vitro cultured neural stem cells—Tools for analyzing dynamics of cell cycle, migration, and lineage selection. Methods.

[9] Svensson C.-M., Medyukhina A., Belyaev I., Al-Zaben N., Figge M.T. (2017). Untangling cell tracks: Quantifying cell migration by time lapse image data analysis. Cytom. Part A.

[10] Mathieu E., Paul C.D., Stahl R., Vanmeerbeeck G., Reumers V., Liu C., Konstantopoulos K., Lagae L. (2016). Time-lapse lens-free imaging of cell migration in diverse physical microenvironments. Lab Chip.

[11] Reverey J.F., Jeon J.-H., Bao H., Leippe M., Metzler R., Selhuber-Unkel C. (2015). Superdiffusion dominates intracellular particle motion in the supercrowded cytoplasm of pathogenic Acanthamoeba castellanii. Sci. Rep..

[12] Fukaya S., Aoki K., Kobayashi M., Takemura M. (2020). Kinetic Analysis of the Motility of Giant Virus-Infected Amoebae Using Phase-Contrast Microscopic Images. Front. Microbiol..

[13] Rudell J., Gao J., Sun Y., Sun Y., Chodosh J., Schwab I., Zhao M. (2013). Acanthamoeba Migration in an Electric Field. Investig. Opthalmol. Vis. Sci..

[14] Campolo A., Shannon P., Crary M. (2021). Evaluating Alternate Methods of Determining the Antimicrobial Efficacy of Contact Lens Care Products against Acanthamoeba Trophozoites. Pathogens.

[15] Crary M., Walters R., Shannon P., Gabriel M. (2021). Variables Affecting the Recovery of *Acanthamoeba* Trophozoites. Pathogens.

[16] Scruggs B.A., Quist T.S., Salinas J.L., Greiner M.A. (2019). Notes from the field: Acanthamoeba keratitis cases—Iowa, 2002–2017. MMWR Morb. Mortal. Wkly. Rep..

[17] Radford C.F., Minassian D.C., Dart J.K.G. (2002). Acanthamoeba keratitis in England and Wales: Incidence, outcome, and risk factors. Br. J. Ophthalmol..

[18] Larkin D.F., Kilvington S., Easty D.L. (1990). Contamination of contact lens storage cases by Acanthamoeba and bacteria. Br. J. Ophthalmol..

[19] Kuburich N.A., Adhikari N., Hadwiger J.A. (2016). Acanthamoeba and Dictyostelium Use Different Foraging Strategies. Protist.

[20] Arnalich-Montiel F., Lumbreras-Fernández B., Martín-Navarro C.M., Valladares B., Lopez-Velez R., Morcillo-Laiz R., Lorenzo-Morales J. (2014). Influence of Acanthamoeba Genotype on Clinical Course and Outcomes for Patients with Acanthamoeba Keratitis in Spain. J. Clin. Microbiol..

[21] Ledee D.R., Iovieno A., Miller D., Mandal N., Diaz M., Fell J., Fini M.E., Alfonso E.C. (2009). Molecular Identification of T4 and T5 Genotypes in Isolates from Acanthamoeba Keratitis Patients. J. Clin. Microbiol..

[22] Maghsood A.H., Sissons J., Rezaian M., Nolder D., Warhurst D., Khan N.A. (2005). Acanthamoeba genotype T4 from the UK and Iran and isolation of the T2 genotype from clinical isolates. J. Med. Microbiol..

[23] TrackMate Algorithims. https://imagej.net/plugins/trackmate/algorithms.

